# Temporal Course of Plasma Trimethylamine N-Oxide (TMAO) Levels in ST-Elevation Myocardial Infarction

**DOI:** 10.3390/jcm10235677

**Published:** 2021-12-01

**Authors:** Mohammad A. Almesned, Femke M. Prins, Erik Lipšic, Margery A. Connelly, Erwin Garcia, Robin P. F. Dullaart, Hilde E. Groot, Pim van der Harst

**Affiliations:** 1 Department of Cardiology, University Medical Center Groningen, University of Groningen, 9713 GZ Groningen, The Netherlands; m.a.m.almesned@student.rug.nl (M.A.A.); f.m.prins@umcg.nl (F.M.P.); e.lipsic@umcg.nl (E.L.); h.e.groot@umcg.nl (H.E.G.); 2 Laboratory Corporation of America Holdings (LabCorp), Morrisville, NC 27560, USA; connem5@labcorp.com (M.A.C.); garce14@labcorp.com (E.G.); 3 Department of Endocrinology, University Medical Center Groningen, University of Groningen, 9713 GZ Groningen, The Netherlands; dull.fam@12move.nl; 4 Department of Cardiology, Division of Heart & Lungs, University Medical Center Utrecht, University of Utrecht, 3584 CX Utrecht, The Netherlands

**Keywords:** trimethylamine N-oxide (TMAO), ST-elevation myocardial infarction (STEMI), infarct size, left ventricular ejection fraction (LVEF), estimated glomerular filtration rate (eGFR)

## Abstract

The gut metabolite trimethylamine N-oxide (TMAO) at admission has a prognostic value in ST-elevation myocardial infarction (STEMI) patients. However, its sequential changes and relationship with long-term infarct-related outcomes after primary percutaneous coronary intervention (PCI) remain elusive. We delineated the temporal course of TMAO and its relationship with infarct size and left ventricular ejection fraction (LVEF) post-PCI, adjusting for the estimated glomerular filtration rate (eGFR). We measured TMAO levels at admission, 24 h and 4 months post-PCI in 379 STEMI patients. Infarct size and LVEF were determined by cardiac magnetic resonance 4 months after PCI. TMAO levels decreased from admission (4.13 ± 4.37 μM) to 24 h (3.41 ± 5.84 μM, *p* = 0.001) and increased from 24 h to 4 months (3.70 ± 3.86 μM, *p* = 0.026). Higher TMAO values at 24 h were correlated to smaller infarct sizes (rho = −0.16, *p* = 0.024). Larger declines between admission and 4 months suggestively correlated with smaller infarct size, and larger TMAO increases between 24 h and 4 months were associated with larger infarct size (rho = −0.19, *p* = 0.008 and rho = −0.18, *p* = 0.019, respectively). Upon eGFR stratification using 90 mL/min/1.73 m^2^ as a cut-off, significant associations between TMAO and infarct size were only noted in subjects with impaired renal function. In conclusion, TMAO levels in post-PCI STEMI patients are prone to fluctuations, and these fluctuations could be prognostic for infarct size, particularly in patients with impaired renal function.

## 1. Introduction

Preventative and therapeutic efforts have contributed to the decline of coronary heart disease (CHD)-related mortality in the past decades. Nevertheless, CHD remains the main global cause of death with rates exceeding 9 million deaths per year, thereby underscoring the importance of furthering our current understanding of this disease [1,2,3].

Atherosclerosis hallmarks CHD, and efforts are currently being directed at better understanding the manner through which this inflammatory process contributes to the development of CHD. Trimethylamine N-oxide (TMAO), a gut microbial product derived from L-carnitine and choline-rich foods, has gained substantial attention due to its atherogenic potential and its impact on CHD prognosis and all-cause mortality [4,5,6,7,8,9]. Indeed, recent endeavors have suggested that higher admission plasma TMAO values reflect a larger atherosclerotic burden and a higher risk of plaque rupture in patients presenting with ST-segment elevation myocardial infarction (STEMI) prior to undergoing percutaneous coronary intervention (PCI) [10,11]. Furthermore, higher systemic TMAO levels exhibited a dose-dependent relationship with increased risk of incident major adverse cardiac events (MACE) in patients presenting with acute coronary syndrome (ACS) [6].

While these aforementioned efforts have certainly delineated the value of pre-PCI TMAO levels in STEMI patients, the relevance of circulating TMAO in the post-PCI context remains elusive. Furthermore, data on the relationship between TMAO and infarct-related outcomes in STEMI patients, which could potentially explain the current established prognostic value of TMAO, is scant. For these reasons, the aim of the current study is to (i) delineate the temporal course of TMAO levels in STEMI patients who underwent primary PCI, and (ii) to outline the link between TMAO levels and infarct-related outcomes 4 months after the occurrence of STEMI.

## 2. Materials and Methods

### 2.1. Study Population and Design

Our study included all patients enrolled in the ‘Glycometabolic Intervention as Adjunct to Primary Coronary Intervention in ST Elevation Myocardial Infarction‘(GIPS-III) trial. This clinical trial aimed to delineate whether metformin treatment preserved left-ventricular ejection fraction (LVEF) in STEMI patients without diabetes [12]. In short, all patients admitted to the University Medical Center Groningen (UMCG) via the STEMI protocol between 1 January 2011 and 26 May 2013 were eligible for inclusion. Further inclusion criteria included an age of older than 18 years, presence of STEMI, and percutaneous coronary intervention (PCI) utilizing a minimum of one stent with a diameter of at least 3 mm which resulted in a post–intervention TIMI flow grade of 2 or 3 [12]. Major exclusion criteria included previous myocardial infarction, known diabetes, the need for coronary artery bypass graft surgery, severe renal dysfunction (creatinine > 177 μmol/L or eGFR < 30 mL/min/1.73 m^2^), and standard contraindications for magnetic resonance imaging (MRI) [12]. The GIPS-III trial showed that the use of metformin had no effect on infarct-related outcomes in STEMI patients after 4 months [12]. Further detail regarding the design and rationale of the GIPS-III trial can be found elsewhere [13]. Patients were treated according to the standard protocol of the UMCG for PCI. Periprocedural medications included heparin, acetylsalicylic acid, and P2Y12 receptor inhibitor. The GIPS-III trial was carried out in agreement with the declaration of Helsinki and approved by the UMCG Medical Ethical Committee (Groningen, the Netherlands, nr: 2010.077); informed consent was obtained from all participating subjects prior to their inclusion [12].

Out of the 1473 STEMI patients admitted to the UMCG between January 2011 and May 2013, 379 subjects befitted the inclusion criteria and were included in our analysis.

### 2.2. Data Collection

Standard physical examination parameters and routine laboratory values were measured at admission as per protocol. Patients returned to the outpatient clinic for follow-up at 2 weeks, 7 weeks, 4 months, and 1 year after discharge. Blood was sampled at these timepoints according to protocol to allow for the routine measurement of common laboratory parameters (i.e., cardiac enzymes) [12,13,14]. Additional blood samples for metabolic profiling (i.e., TMAO) were collected at admission, 24 h after PCI, and at 4 months after PCI and at follow-up points beyond the 4-month mark [12,13]. Samples of sufficient quality for TMAO measurement were only available at admission, 24 h and 4 months after PCI. eGFR was calculated using the Chronic Kidney Disease Epidemiology Collaboration study equation [15]. The total number of subjects after eGFR stratification differs from the total cohort because of missing eGFR data at baseline from some subjects. The methods utilized for MRI measurement of LVEF and infarct size are described elsewhere [13].

Plasma TMAO was quantified using NMR by ^1^H-nuclear magnetic resonance (NMR) spectroscopy employing a Vantera^®^ Clinical Analyzer (LabCorp, Raleigh, NC, USA) as previously reported [16]. To separate TMAO from other trimethylamine-containing species on the NMR spectrum, the pH was lowered to 5.3 by mixing TMAO specimens with a citrate buffer (3:1 *v*/*v*). The Carr-Purcell-Meiboom-Gill (CPMG) acquisition technique was used to obtain NMR spectra, after which TMAO was quantified from TMAO peaks using a non-negative least squares fitting algorithm and subsequently converted to concentration units (μM) through an empirically determined conversion factor. The TMAO assay has intra- and inter-assay coefficients of variation (CV%) that range from 4.3–10.3% and 9.8–14.5%, respectively [17].

### 2.3. Statistical Analysis

Baseline characteristics were expressed as means ± standard deviation when normally distributed, as medians with interquartile ranges when non-normally distributed, or as frequencies and percentages for categorical variables. Differences between groups were determined by Student *t* test, Mann–Whitney *U* test, Chi-square or Fisher’s exact test.

The temporal course of TMAO at admission and in the post-PCI context was studied using a mixed model with random intercepts followed by a pairwise comparison with a Bonferroni correction to delineate differences between specific timepoints. Additionally, we calculated Spearman rank correlation coefficients between TMAO values and infarct-related outcomes at and across the different time points. This analysis was reiterated twice after stratification of subjects based on their admission eGFR values (with 90 and 60 mL/min/1.73 m^2^ as two different cut-off values), seeing that renal impairment has been reported to confound the associations between TMAO and cardiovascular disease [18].

In line with Benjamin et al., a 2-sided *p*-value < 0.005 was considered to be statistically significant, whereas a *p*-value between 0.05 and 0.005 was considered to be suggestive [19]. All analyses were carried out using STATA version 16.0 (StataCorp LLC, College Station, TX, USA). All figures were generated using GraphPad Prism version 9.0.1 (GraphPad Software, LLC, San Diego, CA, USA).

## 3. Results

### 3.1. Study Cohort

Baseline characteristics are presented in Table 1. The median age at presentation was 59 years (interquartile range [IQR] 50.6–66.6 years), and 75% of all subjects were male. The median ischemic time was 161 min (IQR 109–250 min), and the median eGFR was 93 (IQR 80–106 mL/min/1.73 m^2^). Baseline characteristics stratified according to eGFR values at admission with 90 mL/min/1.73 m^2^ as cut-offs are presented in Table 1. The baseline characteristics of groups stratified by eGFR with cut-off point 60 mL/min/1.73 m^2^ can be found in Supplemental Table S1.

### 3.2. Temporal Changes in TMAO

**Figure 1:**
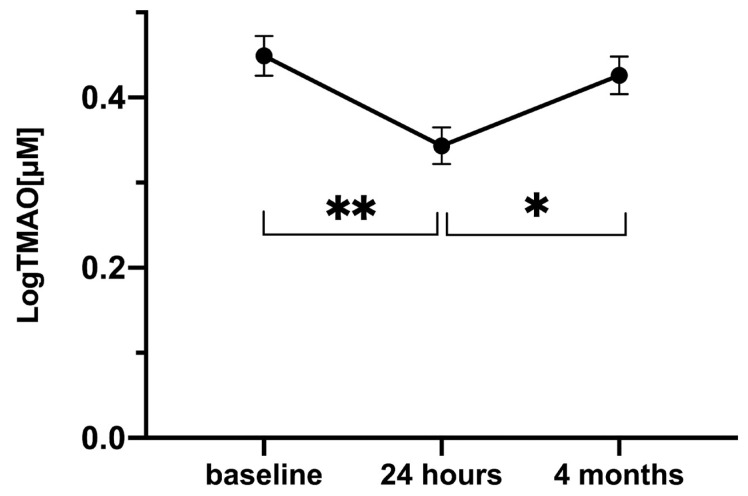
LogTMAO levels across time in subjects admitted via the STEMI protocol (*n* = 379). Mixed model analysis was utilized to contrast the temporal course of logTMAO between categorical groups. Data are presented as mean and standard error of the mean (SEM). * = *p* ≤ 0.05; ** = *p* ≤ 0.001 Abbreviations: TMAO—trimethylamine N-oxide, STEMI—ST-elevated myocardial infarction.

Figure 1 depicts the levels of TMAO at admission, 24 h, and at 4 months after PCI after logarithmic transformation. Mixed model analysis revealed that the mean TMAO concentration differed significantly between the three time points (*p* = 0.001). Post hoc pairwise comparison with an applied Bonferroni correction outlined that the reductions in TMAO levels between admission and 24 h and subsequent increase between 24 h and 4 months were statistically significant (from 0.45 ± 0.02 μM to 0.34 ± 0.02 μM, *p* = 0.001, and from 0.34 ± 0.02 μM to 0.43 ± 0.02 μM, *p* = 0.026, respectively).

### 3.3. TMAO and Infarct-Related Outcomes

Spearman correlation analysis revealed that TMAO levels at the three timepoints, as well as their changes across such timepoints were not associated with LVEF. The gut metabolite did, however, exhibit suggestive associations with infarct size (Table 2). Specifically, higher TMAO levels at 24 h were associated with smaller infarct sizes, whereas larger TMAO declines between admission and 4 months were associated with smaller infarct sizes (rho = −0.16, *p* = 0.024 and rho = −0.19, *p* = 0.008, respectively). Furthermore, larger TMAO increases between 24 h and 4 months were correlated with larger infarct sizes (rho = −0.18, *p* = 0.019) (see Supplementary Table S3 for delta values). The baseline Spearman correlations between TMAO and MRI indices 4 months post-STEMI stratified by eGFR with cut-off point 60 mL/min/1.73 m^2^ can be found in Table S2.

Some of these suggestive findings persisted and became significant after eGFR stratification with 90 mL/min/1.73 m^2^ as a cut-off value, but only in patients with an eGFR < 90 mL/min/1.73 m^2^ (Table 3). To illustrate, in the <90 mL/min/1.73 m^2^ eGFR group, the TMAO decline between admission and 4 months became significantly associated with infarct size (rho = −0.49, *p* < 0.001), and the TMAO increase between 24 h and 4 months became significantly associated with infarct size (rho = −0.35, *p* = 0.004). Additionally, these patients exhibited a positive suggestive correlation between larger increases in TMAO values between admission and 4 months and LVEF (rho = 0.25, *p* = 0.030). Comparing the correlations of the two different groups using a regression model with an interaction term (eGFR × TMAO) showed that the correlations for infarct size suggestively, and significantly, differed for the decline in TMAO levels between admission and 4 months, and the TMAO increase between 24 h and 4 months, respectively (*p* = 0.010; *p* < 0.001), and that the correlations for LVEF suggestively differed for delta TMAO levels between 24 h and 4 months (*p* = 0.013). Lastly, no significant correlations were observed between TMAO values and infarct-related outcomes when subjects were stratified using 60 mL/min/1.73 m^2^ as a cut-off value for eGFR.

## 4. Discussion

We investigated the sequential changes of TMAO and its relationship to infarct-related outcomes before and after adjusting for kidney function in STEMI patients. The major findings of the present study were as follows: (i) TMAO levels significantly decreased within the first 24 h after PCI and returned to baseline values after 4 months, (ii) TMAO exhibited suggestive associations with infarct size but not with LVEF, and (iii) eGFR stratification with 90 mL/min/1.73 m^2^ as a cut-off value accentuated the association between TMAO and infarct size in subjects with eGFR < 90 mL/min/1.73 m^2^.

### 4.1. Admission TMAO Levels and Its Temporal Course

Plasma TMAO levels are prone to influence by an array of factors, including but not limited to gut microbiome composition, dietary choline and L-carnitine intake, and genetic heterogeneity of the hepatic flavin monooxygenase 3 enzyme responsible for the generation of TMAO from trimethylamine [4,20]. Indeed, previous investigations not only showed variation in TMAO levels across different cardiovascular disease cohorts but also across cohorts sharing the same cardiovascular disease pathology [6,21,22]. To illustrate, while investigating the prognostic potential of admission TMAO levels across two ACS cohorts, Xinmin and colleagues observed higher TMAO levels in the Cleveland cohort (median: 4.28 uM) compared to the Swiss cohort (2.87 μM) [6]. Interestingly, our current Dutch cohort (median: 3.1 μM) demonstrated closer affinity to the Swiss cohort admission TMAO level relative to the US cohort. While this observation could be attributed to genetic and/or dietary similarities between our cohort and the Swiss cohort as a result of their geographic proximity relative to the US cohort, we cannot draw such a definitive conclusion since both our study and that of Xinmin et al. lacked information regarding the dietary habits and genetic background of the subjects involved. In terms of studies exclusive to STEMI patients, admission median TMAO levels ranged from 2 μM to 6 μM, a range to which our present STEMI cohort conforms [11,23,24].

Our analysis is the first in (i) delineating changes in TMAO levels across three different timepoints and (ii) reporting such temporal patterns within the first 4 months in STEMI patients who underwent PCI. The drop in TMAO values within the first 24 h of admission in the present cohort indicates that TMAO is prone to acute changes in the post-PCI setting. We could not, however, pinpoint the precise reason behind such variation as subjects were not necessarily fasted, nor were they expected to follow a prespecified dietary regimen upon admission. Analyzing such dietary parameters is of central importance as TMAO has been shown to exhibit volatile changes in response to acute changes in dietary patterns. In a pilot study including healthy participants randomized to a 24 h water-only fast or a 24 h ad libitum fed diet, Washburn et al. reported a significant decrease of TMAO levels at the end of a fasted day, which subsequently returned to admission values within 24 h from the start of the refeeding phase [25].

The only other report describing the sequential change of TMAO after PCI in STEMI patients is that of Matsuzawa et al. In their cohort, TMAO significantly increased between admission (median: 5.63 μM) and 10 months (6.76 μM) [23]. While our present investigation found no significant difference between admission and 4 months, it is important to note that the TMAO change observed by Matsuzawa et al. was borderline significant (or rather suggestive when maintaining our definition of significance; *p* = 0.048). Furthermore, the difference in the temporal period used to measure changes in TMAO levels between their investigation (10 months) and ours (4 months) could also be a reason behind this discrepancy.

### 4.2. TMAO, Long-Term Infarct-Related Outcomes and Renal Function

In addition to the state of post-PCI perfusion, we analyzed the relationship between TMAO and infarct size, as well as LVEF, as both are important predictors of STEMI prognosis [26]. Studies conducted on murine models analyzed the relationship between TMAO and these infarct-related outcomes, but such investigations yielded conflicting results [27,28]. In humans, previous reports showed that TMAO levels were not associated with infarct size as determined by peak admission CK-MB and creatine phosphate values [20,29]. These findings are supported by the recent work of Zhou et al., in which it was shown that baseline TMAO levels did not significantly correlate with infarct size nor with LVEF at 30 days in patients presenting with a first anterior STEMI [30]. Our work extends these findings as it includes all STEMI patients irrespective of the culprit vessel involved and reports the relationship between TMAO (at and across three distinct timepoints) and infarct-related outcomes (at 4 months).

In our study, greater TMAO declines between admission and 4 months reflected smaller infarcts, and greater TMAO elevations between 24 h and 4 months were suggestively correlated to larger infarcts. It is established that the extent of inflammation—a process to which TMAO is linked—in the reperfusion phase is pivotal in determining the size of the infarct [28,31,32]. Such a process ensures the removal of necrotic debris, yet excessive inflammation in itself could expand the area of damage and, subsequently, the size of the infarct [31,32,33]. We hypothesize that greater elevations in TMAO levels between 24 h and 4 months could reflect the persistence of an overactive inflammatory response that spills into, and after, the reparative phase, thereby exacerbating the size of the infarct [33,34].

eGFR stratification showed that such associations became no longer suggestive in patients with normal kidney function (eGFR ≥ 90 mL/min/1.73 m^2^). In the impaired kidney function group (eGFR < 90 mL/min/1.73 m^2^), however, such stratification accentuated some of the suggestive associations into becoming significant, where greater TMAO declines between admission and 4 months became significantly associated with smaller infarcts and greater TMAO elevations between 24 h and 4 months became significantly correlated to larger infarcts. These findings are of relevance in as much as the relationship between TMAO and kidney function is a current topic of discussion. Not only is plasma TMAO mostly cleared by the kidney, but a “uremic environment”—characteristic of CKD patients—may also elevate plasma TMAO levels through promoting dysbiosis of the gut microbiome [35,36]. On the other end of the spectrum, TMAO has been shown to increase tubulointerstitial fibrosis and collagen deposition in murine models [35]. Thus, the question that remains in light of our findings and the nature of our post hoc analysis is whether TMAO’s association with infarct-related outcomes is simply confounded by low eGFR. This possibility is supported by the findings of the aforementioned works of Zhou et al., in which TMAO was not shown to be associated with infarct outcomes, and the work of Sonmez and colleagues, in which decreased eGFR (eGFR < 60 mL/min/1.73 m^2^) was shown to be associated with lower LVEF and greater infarct sizes [30,37]. The lack of significant relationships between TMAO and infarct-related outcomes in our <60 mL/min/1.73 m^2^ eGFR group does not necessarily refute the confounding possibility, seeing that such group comprised of only 14 subjects. In terms of LVEF and its association to TMAO (or changes thereof), we hypothesize that the lack of significant associations between the two variables to be the result of our study population, as well as the limitations associated with using LVEF to represent the myocardial function. GIPS-III subjects underwent rapid PCI, thereby limiting the extent of myocardial infarction and subsequent deterioration of LV function. In doing so, this rapid PCI approach could have limited the variation in LVEF amongst these patients, which could explain the lack of an association between LVEF and TMAO [12,13,14]. Furthermore, inherent to the use of LVEF as a marker of cardiac function and remodeling are physiological, technical, and clinical limitations [38]. In contrast to LVEF, infarct size measurement using MRI allows for a more accurate representation of the functional state of the myocardium and has been shown to be a superior prognostic predictor [39,40]. This could potentially explain why TMAO levels were significantly associated with infarct size but not with LVEF.

### 4.3. Limitations

Several limitations warrant consideration. First, the post hoc nature of our investigation limits our conclusion to being correlative rather than causative. Second, the GIPS-III trial was designed to test metformin in non-diabetic subjects presenting with a first STEMI. In the Dutch system, STEMI patients undergo rapid primary PCI, thereby limiting infarct size and preserving LVEF. Moreover, subjects with severe renal dysfunction at presentation were excluded from the GIPS-III trial. Therefore, our analysis is not free from selection bias, and conclusions should be drawn with caution in light of other STEMI cohorts. Furthermore, we did not prospectively collect information regarding the dietary patterns nor antibiotic use prior to or after PCI, both of which are key in influencing TMAO concentrations [4,41]. However, dietary intake at the coronary care unit (CCU) was standardized according to clinical protocol, and a retrospective review of medical charts revealed that no antibiotics were used by the patients included.

### 4.4. Future Perspectives

The fluctuations in TMAO levels after PCI urge future endeavors to investigate the reasons behind such changes in a controlled environment, taking into account the influence of diet, genetics, and antibiotic use on the gut metabolite. Additionally, more investigations are needed to clarify the relationship between TMAO, eGFR, and infarct-related outcomes.

## 5. Conclusions

In this investigation, we outlined the temporal course of TMAO in the post-PCI context of STEMI patients and associations between TMAO levels and infarct sizes. These associations were accentuated in terms of significance in patients with impaired renal function (eGFR < 90 mL/min/1.73 m^2^), thereby implying an interplay between renal dysfunction, higher TMAO values, and infarct sizes. The baseline Descriptive statistics of post-PCI medication stratified by eGFR with cut-off point 60 mL/min/1.73 m^2^ can be found in Table S4.

## Figures and Tables

**Table 1:** Baseline characteristics of GIPS-III subjects.

			Total	eGFR Groups		
				Reduced	Normal	*p*-Value
				eGFR < 90	eGFR≥90	
			*n* = 379	*n* = 144 *	*n* = 188 *	
Age			59.1 (50.6–66.5)	63.7 (54.6–71.1)	54.9 (48.4–62.7)	<0.001
Female sex			95 (25.1%)	43 (29.9%)	39 (20.7%)	0.056
BMI (kg/m^2^)			26.6 (24.2–29.3)	27.1 (25.1–29.6)	26.2 (24.1–29.1)	0.050
Cardiovascular related history						
	Hypertension		112 (29.6%)	60 (41.7%)	42 (22.3%)	<0.001
	Current smoking		209 (55.1%)	64 (44.4%)	123 (65.4%)	<0.001
	Hypercholesterolemia		239 (63.1%)	83 (57.6%)	126 (67.0%)	0.079
	Stroke		3 (0.8%)	2 (1.4%)	1 (0.5%)	0.580
	Peripheral artery disease		0	0	0	-
	Previous PCI		4 (1.1%)	2 (1.4%)	2 (1.1%)	1.00
Blood Pressure						
	Systolic (mmHg)		133 (120–147)	133 (120–149)	132 (120–146)	0.750
	Diastolic (mmHg)		83 (74–94)	84 (72–93)	83 (76–95)	0.440
Heart rate (beats/min)			74.0 (64.0–85.0)	72.0 (61.0–84.5)	73.0 (65.0–84.0)	0.460
Ischemia time (min)			161 (109–250)	144 (107–203)	170 (113–273)	0.030
Single vessel disease			258 (68.1%)	96 (66.7%)	128 (68.1%)	0.780
Culprit vessel						
	LAD		146 (38.5%)	52 (36.1%)	72 (38.3%)	
	LCX		64 (16.9%)	27 (18.8%)	26 (13.8%)	
	RCA		169 (44.6%)	65 (45.1%)	90 (47.9%)	
Laboratory measures						
	CK-MB (U/L)		16 (13–25)	15 (13–22)	17 (13–27)	0.130
	Troponin (ng/L)		50 (23–136)	49 (26–136)	50 (23–136)	0.970
	NT-proBNP (ng/L)		81 (40–200)	90 (45–218)	77 (37–172)	0.120
	eGFR (mL/min/1.73 m^2^)		93 (80–106)	79 (68–85)	104 (96–117)	<0.001
	TMAO (μM)		3.10 (1.80–5.10)	4.10 (2.50–6.10)	2.80 (1.55–4.00)	<0.001
	Cholesterol (mmol/L)		5.3 (4.7–6.0)	5.2 (4.6–5.9)	5.4 (4.8–6.2)	0.026
	HDL cholesterol (mmol/L)		1.1 (0.9–1.3)	1.1 (0.9–1.3)	1.1 (0.9–1.4)	0.570
	LDL cholesterol (mmol/L)		3.8 (3.2–4.4)	3.6 (3.0–4.3)	3.8 (3.3–4.6)	0.006
	Triglycerides (mmol/L)		0.9 (0.6–1.5)	1.0 (0.7–1.4)	0.9 (0.6–1.6)	0.059

Data are expressed as median (interquartile range [IQR]) or as number (%). *p*-values were based on Student *t* test, Mann–Whitney U test, Chi-square, or Fisher’s exact test when appropriate. * The total number of subjects after eGFR stratification differs from the total cohort because of missing eGFR data at baseline from some subjects. Abbreviations: BMI—body mass index; CK-MB—myocardial band of creatine kinase; eGFR—estimated glomerular filtration rate; PCI—percutaneous coronary intervention; NT-proBNP—N-terminal pro-brain natriuretic peptide; TMAO—trimethylamine N-oxide; LAD—left anterior descending; LCX—left circumflex artery; RCA—right coronary artery; LDL—low-density lipoprotein; HDL—high-density lipoprotein.

**Table 2:** Spearman correlations between TMAO and MRI indices 4 months post-STEMI.

	Infarct Size *		LVEF	
	Rho	*p*-Value	Rho	*p*-Value
TMAO at admission	−0.12	0.064	0.04	0.583
TMAO at 24 h	−0.16	0.024	0.06	0.353
TMAO at 4 months	0.05	0.437	−0.02	0.729
Delta TMAO (admission–24 h)	0.01	0.861	−0.02	0.770
Delta TMAO (24 h–4 months)	−0.18	0.019	0.10	0.163
Delta TMAO (admission–4 months)	−0.19	0.008	0.02	0.726

* Infarct size as % of left ventricular thickness. Abbreviations: TMAO—trimethylamine N-oxide; LVEF—left ventricular ejection fraction.

**Table 3:** Spearman correlations between TMAO and MRI indices 4 months post-STEMI stratified by eGFR.

	Reduced eGFR (eGFR < 90 mL/min/1.73 m^2^) *n* = 144				Normal eGFR (eGFR ≥ 90 mL/min/1.73 m^2^) *n* = 188			
	Infarct Size *		LVEF		Infarct Size *		LVEF	
	**Rho**	***p*** **-Value**	**Rho**	***p*** **-Value**	**Rho**	***p*** **-Value**	**Rho**	***p*** **-Value**
TMAO at admission	−0.28	0.016	0.20	0.068	−0.03	0.779	−0.004	0.968
TMAO at 24 h	−0.20	0.107	0.13	0.244	−0.17	0.081	0.008	0.935
TMAO at 4 months	0.24	0.031	−0.10	0.352	−0.12	0.221	0.09	0.351
Delta TMAO (admission–24 h)	−0.11	0.421	0.08	0.537	0.10	0.312	−0.05	0.638
Delta TMAO (24 h–4 months)	−0.35	0.004	0.19	0.100	0.01	0.914	−0.06	0.575
Delta TMAO (admission–4 months)	−0.49	<0.001	0.25	0.030	0.15	0.122	−0.18	0.059

* Infarct size as % of left ventricular thickness. Abbreviations: TMAO = trimethylamine N-oxide; LVEF = left ventricular ejection fraction; eGFR = estimated glomerular filtration rate.

## Data Availability

The data presented in this study are available on request from the corresponding author.

## Supplementary Materials

The following are available online at https://www.mdpi.com/article/10.3390/jcm10235677/s1, Table S1: Baseline characteristics of 332 GIPS-III subjects stratified by eGFR (cut-off: 60 mL/min/1.73 m^2^). Table S2: Spearman correlations between TMAO and MRI indices 4 months post-STEMI stratified by eGFR (cut-off: 60 mL/min/1.73 m^2^). Table S3: Descriptive statistics of delta TMAO values across all eGFR strata. Table S4: Descriptive statistics of postPCI medication stratified by eGFR (cutoff: 90 mL/min/1.73 m^2^).
