# Supplementary material for: Temporal Course of Plasma Trimethylamine N-Oxide (TMAO) Levels in ST-Elevation Myocardial Infarction
Source: J Clin Med. 2021 Dec 1;10(23):5677. doi: 10.3390/jcm10235677 (PMC8658331; doi:10.3390/jcm10235677)
Supplement: Supplementary file 1 [file jcm-10-05677-s001.zip › jcm-1458139-SI.pdf]

Journal of Clinical Medicine
MDPI

## Supplemental material

Supplementary Table S1: Baseline characteristics of 332 GIPS-III subjects stratified by eGFR (cut-off: 60 ml/min/1.73 m2)

Supplementary Table S2: Spearman correlations between TMAO and MRI indices 4 months post-STEMI stratified by eGFR (cut-off: 60 ml/min/1.73 m2)

Supplementary Table S3: Descriptive statistics of delta TMAO values across all eGFR strata

Supplementary Table S4: Descriptive statistics of postPCI medication stratified by eGFR (cut-off: 90 ml/min/1.73 m2)

**Supplementary Table S1.** Baseline characteristics of 332 GIPS-III subjects stratified by eGFR (cut-off: 60 ml/min/1.73 m2)

| | Total | eGFR groups | | |
|---|---|---|---|---|
| | | Reduced eGFR (eGFR< 60) | Normal eGFR (eGFR ≥ 60) | P-value |
| | n = 332 | n = 14 | n = 318 | |
| **Age** | 59.1 (50.6-66.4) | 72.5 (62.8-80.3) | 58.0 (50.3-65.9) | <0.001 |
| **Female sex** | 82 (24.7%) | 7 (50.0%) | 75 (23.6%) | 0.025 |
| **BMI (kg/m²)** | 26.7 (24.4-29.4) | 29.1 (26.2-31.2) | 26.5 (24.3-29.2) | 0.055 |
| **Cardiovascular related history** | | | | |
| Hypertension | 102 (30.7%) | 10 (71.4%) | 92 (28.9%) | <0.001 |
| Current Smoking | 187 (56.3%) | 4 (28.6%) | 183 (57.5%) | 0.032 |
| Hypercholesterolemia | 209 (63.0%) | 6 (42.9%) | 203 (63.8%) | 0.110 |
| Stroke | 3 (0.9%) | 0 (0.0%) | 3 (0.9%) | 1.00 |
| Peripheral artery disease | 0 | 0 | 0 | - |
| Previous PCI | 4 (1.2%) | 0 (0.0%) | 4 (1.3%) | 1.00 |
| **Blood Pressure** | | | | |
| Systolic (mmHg) | 132 (120-147) | 129 (106-148) | 133 (120-147) | 0.730 |
| Diastolic (mmHg) | 83 (74-95) | 81 (67-87) | 84 (74-95) | 0.210 |
| **Heart rate (beats/min)** | 73.0 (64.0-84.0) | 70.5 (56.0-85.0) | 73.0 (64.0-84.0) | 0.520 |
| **Ischaemia time (min)** | 161 (109-245) | 147 (126-208) | 161 (109-245) | 0.850 |
| **Single vessle disease** | 224 (67.5%) | 10 (71.4%) | 214 (67.3%) | 0.750 |
| **Culprit vessel** | | | | 0.130 |
| LAD | 124 (37.3%) | 2 (14.3%) | 122 (38.4%) | |
| LCX | 53 (16.0%) | 2 (14.3%) | 51 (16.0%) | |
| RCA | 155 (46.7%) | 10 (71.4%) | 145 (45.6%) | |
| **Laboratory measures** | | | | |
| CK-MB (U/L) | 16 (13-24) | 15 (13-19) | 16 (13-24) | 0.640 |
| Troponin (ng/L) | 50 (25-136) | 73 (30-137) | 50 (25-133) | 0.460 |
| NT-proBNP (ng/L) | 83 (40-195) | 246 (89-627) | 80 (40-180) | 0.036 |
| eGFR (ml/min/1.73 m²) | 93 (80-106) | 50 (47-57) | 94 (82-106) | <0.001 |
| TMAO (μM) | 3.10 (1.80-5.00) | 6.30 (5.10-8.95) | 3.10 (1.80-4.80) | <0.001 |
| Cholesterol (mmol/L) | 5.3 (4.7-6.0) | 4.6 (4.1-5.2) | 5.3 (4.8-6.0) | 0.008 |
| HDL-cholesterol (mmol/L) | 1.1 (0.9-1.3) | 1.1 (0.9-1.2) | 1.1 (0.9-1.3) | 0.440 |
| LDL-cholesterol (mmol/L) | 3.8 (3.2-4.4) | 3.2 (2.6-3.8) | 3.8 (3.2-4.4) | 0.017 |
| Triglycerides (mmol/L) | 0.9 (0.6-1.5) | 1.0 (0.7-1.6) | 0.9 (0.6-1.4) | 0.610 |

Data are expressed as mean ± standard deviation (SD), median (interquartile range [IQR]), or as number (%). P-values were based on Student t test, Mann–Whitney U test, Chi-square or Fisher's exact test when appropriate. Abbreviations: BMI = body mass index; CK-MB = myocardial band of creatine kinase; eGFR = estimated glomerular filtration rate; PCI = percutaneous coronary intervention; NT-proBNP = N-terminal pro brain natriuretic peptide; TMAO = trimethylamine N-oxide; LAD = left anterior descending; LCX = left circumflex artery; RCA = right coronary artery; LDL = low-density lipoprotein; HDL = high-density lipoprotein.

**Supplementary Table S2.** Spearman correlations between TMAO and MRI indices 4 months post-STEMI stratified by eGFR (cut-off: 60 ml/min/1.73 $m^2$).

| | **Reduced eGFR (eGFR < 60 ml/min/1.73 $m^2$) (n=14)** | | | | **Normal eGFR (eGFR ≥ 60 ml/min/1.73 $m^2$) (n = 318)** | | | |
|---|---|---|---|---|---|---|---|---|
| | **Infarct size** | | **LVEF** | | **Infarct size** | | **LVEF** | |
| | **Rho** | **P-value** | **Rho** | **P-value** | **Rho** | **P-value** | **Rho** | **P-value** |
| TMAO at admission | -0.49 | 0.320 | 0.54 | 0.266 | -0.11 | 0.130 | 0.06 | 0.431 |
| TMAO at 24 hours | 0.06 | 0.913 | -0.03 | 0.957 | -0.18 | 0.021 | 0.04 | 0.549 |
| TMAO at 4 months | 0.05 | 0.935 | -0.20 | 0.747 | 0.03 | 0.723 | 0.02 | 0.752 |
| Delta TMAO (admission - 24h) | -0.62 | 0.269 | 0.90 | 0.037 | 0.05 | 0.533 | -0.04 | 0.639 |
| Delta TMAO (24 h – 4 months) | 0.50 | 0.391 | -0.21 | 0.741 | -0.17 | 0.041 | 0.06 | 0.444 |
| Delta TMAO (admission - 4 months) | -0.63 | 0.368 | 0.80 | 0.200 | -0.13 | 0.091 | -0.001 | 0.991 |

Infarct size as % of left ventricular thickness. Abbreviations: TMAO = trimethylamine N-oxide; LVEF = left ventricular ejection fraction; eGFR = estimated glomerular filtration rate.

**Supplementary Table S3:** Descriptive statistics of delta TMAO values across all eGFR strata

| | **Total n = 379** | **eGFR < 60 n = 14** | **eGFR ≥ 60 n = 318** | **eGFR < 90 n = 144** | **eGFR ≥ 90 n = 188** |
|---|---|---|---|---|---|
| Delta TMAO (admission - 24h) | 0.27 ± 0.04 | 0.67 ± 0.07 | 0.26 ± 0.04 | 0.36 ± 0.6 | 0.22 ± 0.06 |
| Delta TMAO (24 h – 4 months) | 0.15 ± 0.06 | 0.44 ± 0.49 | 0.15 ± 0.06 | 0.10 ± 0.10 | 0.20 ± 0.09 |
| Delta TMAO (admission - 4 months) | 0.26 ± 0.04 | 0.81 ± 0.15 | 0.24 ± 0.04 | 0.45 ± 0.06 | 0.12 ± 0.06 |

Data is presented as mean ± standard error (SE). Abbreviations: eGFR = estimated glomerular filtration rate; TMAO = trimethylamine N-oxide.

**Supplementary Table S4:** Descriptive statistics of post-PCI medication stratified by eGFR (cut-off: 90 ml/min/1.73 m2).

| | Total subjects | Total subjects with eGFR measurements available at baseline | eGFR groups | | |
|---|---|---|---|---|---|
| | | | Reduced eGFR < 90 | Normal eGFR ≥ 90 | |
| | **n = 379** | **n = 332** | **n = 144** | **n = 188** | **P-value** |
| Aspirin | 367 (96.8%) | 322 (97.0%) | 141 (97.9%) | 181 (96.3%) | 0.52 |
| Clopidogrel | 270 (71.2%) | 238 (71.7%) | 97 (67.4%) | 141 (75.0%) | 0.13 |
| Prasugrel | 4 (1.1%) | 4 (1.2%) | 2 (1.4%) | 2 (1.1%) | 1.00 |
| Ticagrelor | 105 (27.7%) | 90 (27.1%) | 45 (31.2%) | 45 (23.9%) | 0.14 |
| Thienopyridine | 379 (100.0%) | 332 (100.0%) | 144 (100.0%) | 188 (100.0%) | - |
| Coumarine | 20 (5.3%) | 18 (5.4%) | 7 (4.9%) | 11 (5.9%) | 0.81 |
| Beta-blocker | 362 (95.5%) | 315 (94.9%) | 138 (95.8%) | 177 (94.1%) | 0.62 |
| ACE-inhibitor | 301 (79.4%) | 261 (78.6%) | 113 (78.5%) | 148 (78.7%) | 0.96 |
| Calcium-channel blocker | 12 (3.2%) | 12 (3.6%) | 4 (2.8%) | 8 (4.3%) | 0.56 |
| Aldosterone receptor antagonist | 38 (10.0%) | 32 (9.6%) | 13 (9.0%) | 19 (10.1%) | 0.74 |
| Diuretic | 12 (3.2%) | 12 (3.6%) | 7 (4.9%) | 5 (2.7%) | 0.38 |
| Statin | 377 (99.5%) | 330 (99.4%) | 143 (99.3%) | 187 (99.5%) | 1.00 |
| Insulin | 5 (1.3%) | 3 (0.9%) | 1 (0.7%) | 2 (1.1%) | 1.00 |
| Oral antihyperglycaemic | 4 (1.1%) | 2 (0.6%) | 1 (0.7%) | 1 (0.5%) | 1.00 |

Data shown as count The total number of subjects after eGFR stratification differs from the total cohort because of missing eGFR data at baseline from some subjects (%). P-values were based on Student t test, Mann–Whitney U test, Chi-square or Fisher's exact test when appropriate. **Abbreviations**: eGFR = estimated glomerular filtration rate; PCI = percutaneous coronary intervention.
